# Reduction of Luxations of the Thigh

**Published:** 1842-03

**Authors:** 


*Reduction of Luxations of the Thigh.*—The Journal of the Medical Society of Montpelier, contains a memoir on a new method of of reducing luxations of the thigh. This method was first proposed by M. Collin in an inaugural thesis presented to the Faculty of Medicine at Montpelier in 1832, and is now advocated by M. Jaumes, of the same place, who gives some very striking cases. We content ourselves with giving a sketch of the plan; its simplicity commends it to a trial, at least, and if it be found as useful in other hands as in those of M. Collin, he will certainly have conferred a benefit upon science.

It is first necessary to put the patient on a plank strong enough to sustain the weight of the body; the width should be about two feet, the length somewhat to exceed that of the trunk; in this plank four holes are to be bored near the four corners, through which cords are to be passed so as to suspend it about four feet from the ground; the cords, at one end, to be six inches shorter than those at the other, so that one end of the plank shall be six inches higher than the other. This plank being covered with a thin, firm mattrass or other like bedding, the patient is to be placed upon it. flat on his belly, the arms to hang down on either side, the pelvis situated at the highest end of the plank, rests upon the anterior superior illiac spines, the lower extremities hang over the end of the plank so as to form an angle slightly acute with the body. The patient should be allowed to remain in this position a short time without any efforts being made by way of traction on the dislocated limb; the position alone, merely by the weight of the limb, in one of M. Collins cases, restored the dislocated bone. If this does not happen, slight traction may be made *directly downwards*; if this fail, weights may be attached to the limb, a towel being tied round the ankle, and forming a bag beneath the foot, into which weights may be placed. Such is the simple apparatus and the mode of using it, by which M. Collin asserts that he has reduced several cases; one that had resisted extension and counter extension for hours. The plan will, of course, only apply to luxations backwards and upwards on the dorsum of the ileum. M. Jaumes enters into a long argument to prove the reasonableness of this plan; this we shall not translate. The plan has been tried and succeeded; it is simple and painless; let it be tried again,—*N. Y. Med. Gazette.*

